# Knowing isn’t doing: a behavior change perspective on chronic pain management

**DOI:** 10.3389/fpubh.2026.1876192

**Published:** 2026-08-26

**Authors:** Janet R. Bezner

**Affiliations:** Department of Physical Therapy, Texas State University, Round Rock, TX, United States

**Keywords:** behavior change, health promotion, pain management (MeSH), pain science education, self-management

## Abstract

Chronic pain management increasingly emphasizes patient self-management behaviors such as regular physical activity, restorative sleep, stress regulation, and healthful nutrition. Education is commonly used to support these aims; however, improving knowledge alone rarely produces sustained behavior change. In this Perspective, an argument will be made that effective chronic pain management requires a deliberate shift from education-focused approaches alone toward clinician-supported behavior change. Education may be necessary to reconceptualize pain and reduce perceived threat, though it is insufficient to help patients consistently adopt and sustain health-promoting behaviors in the context of pain. Drawing on pain science, health behavior change theory, and behavioral medicine, behavior change will be distinguished from education, and practical techniques will be outlined that clinicians can use to support patients in translating knowledge into action. Key principles include tailoring interventions to a patient’s stage of change, fostering self-efficacy through graded mastery experiences, using motivational interviewing to elicit values-based motivation and address ambivalence, and employing collaborative goal setting and action planning. The role of action planning, graded tasks, and motivational interviewing approaches to sustain behavior change will be emphasized. This paper will provide a clinician-focused roadmap for supporting behavior change in pain care. By moving beyond education toward intentional behavior change support, clinicians can better foster resilience, functional recovery, and sustainable self-management among individuals living with chronic pain.

## Introduction

The recognition that “pain” is not a reliable indicator of the state of body tissues (1), and that pain is a biopsychosocial experience (2), transformed chronic pain management and contributed to the development of pain neuroscience education and pain science education, or explaining pain to patients with chronic pain (3). Pain science education (PSE) is a major aspect of chronic pain management adopted by health providers like physiotherapists and physicians, who commonly work with a large proportion of patients with chronic pain. The effectiveness of education about pain has been studied by many researchers (2, 3), yielding mixed results and leading to calls for more rigorous research, accurate labeling of the education being provided, and improved competency of the clinicians providing the education. While these explanations of the failure of education about pain to produce consistent positive health outcomes are justified, little has been written about the inadequacy of education *alone* to produce behavior change in patients with chronic pain (4). When intervention consists only of education about pain, advice giving, and/or prescriptive self-management, patients are not well served.

A substantial body of evidence from systematic reviews and meta-analyses demonstrates that regular physical activity, restorative sleep, stress-regulation strategies, and healthful dietary patterns are core, nonpharmacologic components of effective chronic pain management (5–10). Therefore, the provision of clinician-supported behavior change seems as important, if not more important, than education alone in producing meaningful positive health outcomes in patients with chronic pain (11). Contemporary clinical practice guidelines for chronic musculoskeletal pain consistently recommend incorporating education, self-management support, physical activity, and other biopsychosocial and health behavior change interventions into routine care. However, implementation of these recommendations across health professions remains incomplete (12–14). In this perspective, the author will provide a clinician-focused roadmap for supporting behavior change in pain management. Moving beyond education toward intentional behavior change support will enable clinicians, namely physical therapists, occupational therapists, physicians, and chiropractors, to better foster resilience, functional recovery, and sustainable self-management among individuals living with chronic pain.

### Identifying a target behavior

Many of the self-management approaches demonstrated to improve quality of life and health outcomes are behaviors that patients must adopt and sustain, including regular physical activity, high quality sleep, management of stress, and healthful eating. Developing a systematic approach to identify the behavior or behaviors patients are most interested in practicing, and adopting evidence-based strategies will provide useful tools for clinicians to support patients’ behavior change journeys.

Assuming clinicians will provide education about pain as a first step in an intervention plan for chronic pain management, a logical place to start is to weave behavior change conversations into education. For example, a discussion about ‘calming nerves’ or strategies to increase the brain’s production of chemicals that decrease pain, can lead to a conversation about the patient’s interest in pursuing self-management techniques that calm nerves, such as aerobic exercise and sleep hygiene. Another opportunity occurs when discussing the stress response to pain and the role of the sympathetic nervous system. The clinician can explain the contribution of regular health behaviors to dampening the sympathetic nervous system and inquire about the patient’s interest in one or more of these behaviors to mediate stress.

The first step towards successful behavior change is identifying patient interest and motivation in a specific behavior. Clinicians can simply ask the patient if there is a health behavior they are interested in changing, providing examples if necessary to spur patient response. An agenda setting chart can be used to identify a behavior a patient is interested in changing (15, 16). This visual tool displays images and associated descriptors of common health behaviors a patient could adopt that would support self-management of pain and improve overall health. Once the clinician senses patient interest in a health behavior, permission should be requested to talk further about the behavior. This approach ensures the patient retains autonomy in the therapeutic relationship, a key principle for sustaining behavior change (17).

### Principles for supporting behavior change

Effective behavior change support in chronic pain management requires clinicians to move beyond education toward autonomy-supportive, confidence-building, and context-sensitive strategies that recognize and resolve ambivalence, honor patient experience, and translate knowledge into sustained action. The key principles for supporting behavior change have been well established and include respecting and supporting patient autonomy, identifying and supporting patient motivation, enhancing self-efficacy, implementing stage-matched interventions, identifying and managing patient resistance, leading with curiosity rather than judgment, developing a partnership with the patient, and normalizing relapse (Table 1). These principles are rooted in existing behavior change theories and intervention approaches, including self-determination theory, the transtheoretical model, social cognitive theory, motivational interviewing, health coaching, the fear-avoidance model, the health action process approach, acceptance and commitment therapy, trauma-informed care, compassion-focused approaches, the behavior change taxonomy, and the relapse prevention model (18–25).

**Table 1:** Core principles for clinician-supported behavior change in chronic pain management.

Behavior change principle	Description in clinical practice	Example clinician behaviors	Key theories/frameworks
Support patient autonomy	Respect patients’ right to choose whether, when, and how to change behavior. Avoid coercion and emphasize choice.	Offer multiple options rather than a single directive Ask permission before providing advice Emphasize patient choice using autonomy-supportive language (e.g., “could” vs. “should”)	Self-determination theory; motivational interviewing
Elicit motivation rather than prescribe change	Draw out patients’ own reasons for change by exploring personal values and goals rather than emphasizing clinician priorities.	Ask open-ended questions (e.g., “What matters most to you right now?”) Reflect patient values and desires Avoid “should” or “must” language	Motivational interviewing; self-determination theory; transtheoretical model
Enhance self-efficacy through mastery experiences	Build confidence by creating opportunities for achievable success through graded, individualized steps.	Use graded activity or pacing strategies Celebrate small successes Reinforce patients’ ability to act despite pain	Social cognitive theory; fear-avoidance model
Match strategies to readiness for change	Tailor counseling and intervention strategies to the patient’s current stage of change rather than assuming readiness.	Explore readiness before action planning Normalize ambivalence in early stages Delay prescriptive planning until readiness increases	Transtheoretical model; health action process approach
View resistance as meaningful information	Interpret hesitation, avoidance, or non-adherence as insights into beliefs, fears, or contextual barriers rather than noncompliance.	Respond to resistance with reflection and empathy rather than correction Ask curious follow-up questions Adjust approach based on expressed concerns	Motivational interviewing; Acceptance and Commitment Therapy (ACT)
Adopt a curious, non-judgmental stance	Maintain empathy, openness, and psychological safety, particularly during setbacks or lapses.	Use reflective listening Normalize struggle and setbacks Avoid blame, shame, or moral framing	Motivational interviewing (Spirit); trauma-informed care; compassion-focused approaches
Translate knowledge into action collaboratively	Pair education with goal setting, action planning, and problem solving tailored to patient context and capacity.	Co-create SMART goals Develop clear action plans (what, when, how) Anticipate barriers and plan adaptations	Behavior change technique taxonomy; social cognitive theory
Normalize lapses and support maintenance	Frame setbacks as expected, focusing on learning, adaptation, and long-term persistence rather than short-term success.	Prepare patients for lapses in advance Reframe setbacks as learning opportunities Reinforce ongoing self-regulation and flexibility	Relapse prevention models; ACT

SMART, specific, measureable, action-oriented, relevant, and time-referenced.

While not new, the application of these theories and approaches to chronic pain management is not well established and is being emphasized in this manuscript as a result. The principles can be organized into two categories: (1) clinician attitude and communication, and (2) intervention approaches. Regarding clinician attitude and communication, the most important shift a clinician can make in all contexts of provider-patient interaction to support patient behavior change is to adopt a partnership approach vs. an expert approach. While clinicians are experts in their practice arenas, behavior change is a highly individualized and context-dependent experience, and patients are generally better equipped to plan and manage their own behavior change than clinicians. Respecting and supporting patient autonomy includes providing options rather than prescriptions, asking permission before giving advice, and avoiding the use of coercive language and threats. Rather than telling patients what they “should” do, clinicians should evoke patients’ own reasons for change by exploring values, goals, and perceived benefits (through the use of motivational interviewing, for example). Patients are more likely to initiate and maintain behavior change when motivation is intrinsic rather than externally imposed, especially in the context of pain, where ambivalence is common (26). When patients express reluctance, resistance, or avoidance, clinicians should recognize these responses as meaningful information about beliefs, fears, prior experiences, or perceived barriers, rather than patient failure. Countering patient resistance with evidence, argument, shame, or blame typically creates more resistance, as patients defend their autonomy. A more productive response includes empathy, compassion, and curiosity for the patients’ beliefs and experiences, and/or lack of readiness to take the next step. Being curious about the patient and their experiences and beliefs builds and protects the therapeutic alliance and avoids judgment, as it is difficult to be curious and judge at the same time (27).

The intervention approach principles include intentionally enhancing self-efficacy, or the confidence to engage in a behavior, which is a strong predictor of behavior initiation and persistence, and problem-solving to overcome barriers. Clinicians should deliberately design experiences that help patients succeed at manageable steps (e.g., graded activity, small achievable goals, etc.), reinforcing confidence in their ability to act despite pain. A second intervention principle involves assessing stage of change for the behavior and matching interventions to the specific stage (e.g., precontemplation, contemplation, preparation, action, maintenance) (28). Interventions that are mismatched to readiness (e.g., action-oriented plans for ambivalent patients) often provoke resistance and disengagement rather than progress. Capitalizing on respect for autonomy, developing interventions in collaboration with the patient reinforces motivation and empowerment. Collaborative goal setting, action planning, and problem solving ensure that patient experiences are considered, and the interventions fit into the reality of the patient’s current life situations. The principles supporting behavior change are mapped to the theories and frameworks that support them, along with examples of clinician behaviors that demonstrate the principles in Table 1. While formal training in motivational interviewing and Acceptance and Commitment Therapy (ACT), for example, may benefit clinicians, adherence to the principles outlined is consistent with these approaches and thus will support patient behavior change. Applying the principles of behavior change in the implementation of behavior change strategies throughout an episode of care will support patients in developing sustainable health behaviors to manage their pain.

### Key behavior change strategies

Numerous behavior change strategies have been developed and promoted based on the multitude of behavior change theories that have been developed. Researchers have investigated which behavior change techniques are most effective in producing behavior change in patients with various diagnoses. The research in patients with chronic pain is more limited, though some conclusions are helpful in guiding clinician practice (29–33).

Michie et al. (23) created a behavior change taxonomy (BCT) that categorizes the various behavior change strategies or techniques. Using the BCT to categorize techniques in a scoping review, Soderlund et al. (29) identified “information of natural consequences,” “feedback and monitoring,” and “goals and planning” to be the most common behavior change technique categories used in studies on musculoskeletal pain. The specific techniques included in these three categories were providing information on health consequences; providing feedback on the behavior, self-monitoring of the behavior, and on the outcome of the behavior; and action planning, problem solving/coping planning, goal setting for the outcome and behavior, and reviewing goals, respectively. Burke et al. (32), studying interventions utilized to improve long-term adherence to rehabilitation (including patients with chronic low back pain), found that the behavior change techniques typically used are those that reflect common practice, such as goal setting and instructions about how to perform a behavior. Pouliopoulo et al. (33) found somewhat different results to Soderlund et al. (29) and Burke et al. (32) in their scoping review to document the behavioral components of joint protection programs for hand osteoarthritis. For hand osteoarthritis, the most common specific behavioral change techniques used were instruction on how to perform the behavior, behavior substitution, and restructuring the physical environment. A conclusion common to all three of these studies is that a systematic, theory-based approach is seldom evident in research studies examining support for behavior change in patients experiencing pain, and only a small number of BCTs are utilized (29, 32, 33). Evidence is lacking to indicate which BCTs are most effective to support behavior change in patients with pain (31, 32), and whether if more BCTs were used, positive outcomes may occur (29). Examples of evidence-based behavior change techniques, their application in chronic pain management, and references can be found in Table 2. The behavior change techniques can be applied in any setting or context in which a clinician encounters a patient who desires to change a behavior or is willing to explore finding the motivation to change a behavior. Further, it is not uncommon to use multiple BCTs with a patient because one or more may not be helpful, or the patient’s situation changes and requires a different technique or approach. The clinician is best served by considering the techniques in Table 2 as options that can be tried rather than a rigid template they should implement.

**Table 2:** Evidence-supported behavior change techniques in chronic pain management and clinical implications.

Behavior change technique (BCTTv1)	Example in chronic pain management	Implications for clinicians	Key references
Goal setting (behavior)	Collaboratively set specific, meaningful goals for activity, sleep, or stress regulation that are independent of pain levels (e.g., time-contingent walking goals)	Shift from prescribing targets to *co-creating goals* that align with patient values; prioritize functional and participation-focused goals over pain reduction alone	(23, 29, 45, 46)
Action planning	Develop explicit plans that specify when, where, and how behaviors (e.g., activity, pacing, relaxation) will be performed	Pair education with concrete planning; ensure patients leave visits with a clear, feasible action plan rather than general advice	(23, 45)
Self-monitoring of behavior	Use activity logs, step counts, sleep diaries, or digital tools to track behavior patterns over time	Encourage awareness of behavior rather than symptom monitoring alone; use tracking to support learning, not surveillance or compliance	(23, 47)
Feedback on behavior	Review tracked activity, pacing, or sleep data together to reinforce progress and adjust plans	Frame feedback as collaborative reflection; emphasize progress and learning rather than judgment or correction	(23, 45)
Graded tasks	Gradually increase activity or exposure to reduce fear-avoidance (e.g., graded walking, lifting, or daily activities)	Use graded progression deliberately to build confidence and tolerance; avoid pain-contingent progression that reinforces fear and avoidance	(23, 46)
Problem solving	Identify anticipated barriers (e.g., pain flares, fatigue, time constraints) and collaboratively developing coping strategies	View setbacks as expected; dedicate time to barrier identification and adaptation rather than escalating instruction or advice	(23, 46)
Instruction on how to perform the behavior	Teach pacing strategies, relaxation techniques, or movement adaptations with an emphasis on practice and application	Provide instruction as a supporting skill, not a standalone intervention; revisit and refine techniques over time	(23, 31)
Reducing avoidance/exposure to feared activities	Encourage engagement in valued but avoided activities using time-contingent or values-based exposure	Reorient care away from protection and avoidance toward safe engagement and participation; explicitly address fear-avoidance beliefs	(22, 46)
Social support (practical and emotional)	Involve peers, family members, or clinicians to provide encouragement, accountability, and normalization of challenges	Recognize behavior change as socially embedded; leverage support networks to enhance persistence and resilience	(23, 47)
Motivational interviewing–consistent techniques	Explore ambivalence, elicit patient-defined reasons for change, and reinforce autonomy in decision-making	Replace persuasive or directive communication with curiosity-based dialogue; treat resistance as information, not nonadherence	(48, 49)
Relapse prevention/coping planning	Anticipate lapses (e.g., pain flares, life stressors) and plan adaptive responses to sustain behavior change	Normalize non-linear change; support long-term self-management by preparing patients for fluctuations rather than seeking perfect adherence	(23, 31)

### Example of a behavior change conversation

Clinicians are trained to use their expertise to improve patient outcomes. Shifting from an “expert” approach to a “partner” or “coach” approach can be challenging and may feel unnatural. The reality of practice is that clinicians may function as an expert and a partner or coach in the same visit. Knowing when to play each role is important to provide the support patients need to be successful. Table 3 contains an example of an exchange between a clinician provider and a patient, illustrating a behavior change conversation in the context of treating chronic pain. The principles and behavior change techniques included in the conversation are noted in the table. Effective behavior change language, like motivational interviewing, is much less directive than clinician expert language, necessitating clinician intention and practice to effectively support patient behavior change.

**Table 3:** Example clinician-patient conversation illustrating behavior change principles and techniques.

Clinician statement	Patient response	Behavior change principle	Behavior change technique
“When you have had pain for a long time, your nervous system can start acting like it’s on high alert all the time. Nothing is wrong with your nerves, they are doing their job *too* well. They’ve learned that your body might be at risk, so they respond quickly and loudly. When we talk about “calming the nerves,” what we are doing is helping your system realize you are safer than it thinks.”	“How do I calm my nerves?”	N/A	N/A
“There are several ways you can calm your nerves: movement that your body can trust, breathing, sleep, reducing stress, and gradually doing things that matter to you again.”	“I definitely do not sleep very well, and I think it would help me to sleep more and better. I feel tired a lot.”	N/A	N/A
“Would you like to talk about strategies to improve your sleep?”	“Yes”	Support patient autonomy	N/A
“What is your current sleep pattern?”	“I can go to sleep most nights, and I sleep for about 4 h then I wake up. I start thinking about things like my pain and what I cannot do and cannot go back to sleep.”	Elicit motivation rather than prescribe change (asking open ended questions)	N/A
“That sounds frustrating. Feeling tired can make everything seem more difficult.”	“It does! And, I do not feel motivated I think because I’m tired.”	Adopt a curious, non-judgmental stance; empathize	N/A
“How would you like to improve your sleep?”	“Before I was dealing with constant pain, I slept 7–8 h per night and I felt rested when I woke up. If I could consistently get 7 or more hours of sleep each night, that would be great.”	Support patient autonomy; adopt a curious, non-judgmental stance	Goal setting
“It sounds like getting more sleep would help you have more energy to do other things you have said are important, including decreasing your pain. Would you like to set a goal this week to start improving your sleep?”	“It sounds good, but I’m not sure I can do anything about it as long as I’m dealing with this pain.”	Elicit motivation rather than prescribe change; empathize	Goal setting
“I understand you have concerns about your ability to actually change your sleep habits right now. On a scale of 1–10, 1 being not ready at all and 10 being absolutely ready to change, how ready are you to change your sleep habits today?”	“I would say I am a 6/10.”	View resistance as meaningful information; match strategies to readiness to change	Motivational interviewing consistent techniques
“Tell me what makes you a 6/10 and not a 2 or 3/10?”	“I have slept 7–8 h for a lot of my adult life consistently, so I know I can do it.”	Support patient autonomy; adopt a curious, non-judgmental stance; elicit motivation rather than prescribe change; match strategies to readiness to change	Motivational interviewing consistent techniques
“So you have done it before and that experience is helpful. What would it take to be an 8/10 or 9/10 on the readiness scale?”	“I’m not sure, I cannot see past the pain, which tends to wake me up every night and keep me awake.”	Match strategies to readiness to change	Motivational interviewing consistent techniques
“You are seeing the pain as a barrier to getting more sleep. Do you want to try to find a strategy you could start applying this week to see if you can sleep longer or go back to sleep if you wake up?”	“Sure, if you have ideas about something I could try, I’m all ears.”	Support patient autonomy; adopt a curious, non-judgmental stance; elicit motivation rather than prescribe change; translate knowledge into action collaboratively	Action planning; problem solving
“I have a “what to do at 2:00 a.m.” checklist we can review together to identify options you could consider. Are you interested in doing that?”	“Yes”	Support patient autonomy; elicit motivation rather than prescribe change	Action planning; problem solving
“Here is the checklist. Look over it and let me know which of the strategies you want to start with.”	“When I wake up my thoughts immediately go to my pain, so I think one of the “calming the nerves” practices would be useful. Can you teach me the slow breathing technique?”	Support patient autonomy; elicit motivation rather than prescribe change	Action planning; problem solving
(Clinician teaches slow breathing techniques). “Now that you have learned a slow breathing technique you can use when you wake up at night, are you ready to set a goal to practice it this week?”	“Yes”	Support patient autonomy; elicit motivation rather than prescribe change; translate knowledge into action collaboratively	Action planning; instructions on how to perform the behavior; graded tasks
“What would you like the goal to be? For example, trying the breathing technique a specific number of times this week? Or keeping track of when you use the breathing technique and the impact it has on your ability to return to sleep?”	“I would like to try the breathing technique at least 3 times this week when I wake up at night. I will make some notes when I wake up so I can track the effect on my sleep.”	Support patient autonomy; elicit motivation rather than prescribe change; translate knowledge into action collaboratively	Action planning; graded tasks; self-monitoring of behavior
“Sounds like the goal is ‘I will practice slow breathing when I wake up in the middle of the night at least 3 times this week.’ A second goal could be ‘I will keep track of when I use the slow breathing technique by writing down notes and impact when I wake up in the morning.’ Do those 2 goals sound appropriate?”	“Yes”	Support patient autonomy; elicit motivation rather than prescribe change; translate knowledge into action collaboratively	Action planning; graded tasks; self-monitoring of behavior
“On a scale of 1–10, with 1 being not confident at all and 10 being absolutely confident, how confident are you that you will use the slow breathing technique 3 times this week?”	“I am a 7/10 in confidence. I think I can try it.”	Enhance self-efficacy through mastery experiences	Motivational interviewing consistent techniques
“Sounds like you have a plan. Do you have any questions about what you are going to focus on this week?”	“No, I’m looking forward to seeing if this helps me get more sleep.”	N/A	N/A

## Discussion

Researchers have established that engagement in regular behaviors such as physical activity, restorative sleep, stress regulation, and healthful nutrition is an effective approach to reducing and managing chronic pain (5–10). At the population level, adherence to foundational health behaviors remains low among U.S. adults. Fewer than one-third meet combined physical activity guidelines, over one-third report insufficient sleep, the majority report significant unmet stress and emotional support needs, and fewer than 1 in10 eat the recommended amount of vegetables (34–41). These data highlight a substantial implementation gap between evidence-based recommendations and real-world behavior in adults, and this gap is even more pronounced among individuals with chronic pain, among whom only about 18% meet physical activity guidelines, up to 67–88% experience sleep disturbance, and unhealthy dietary patterns are highly prevalent (42–44). Investment in strategies to train clinicians who treat patients with chronic pain in behavior change approaches is therefore warranted. This manuscript provides practical principles and strategies to guide clinicians to support patient behavior change in their practice to best serve patients with chronic pain. Patient performance of regular self-maintenance behaviors, such as physical activity, sleep, stress management, and healthful nutrition, will better foster resilience and functional recovery among individuals living with chronic pain.

## Data Availability

The original contributions presented in the study are included in the article/supplementary material, further inquiries can be directed to the corresponding author.
